# Eco-Friendly Extraction of Phlorotannins from *Padina pavonica*: Identification Related to Purification Methods Towards Innovative Cosmetic Applications

**DOI:** 10.3390/md23010015

**Published:** 2024-12-28

**Authors:** Moustapha Nour, Valérie Stiger-Pouvreau, Alain Guenneguez, Laurence Meslet-Cladière, Stéphane Cérantola, Ahmed Ali, Gaelle Simon, Abdourahman Daher, Sylvain Petek

**Affiliations:** 1 Univ Brest, Institut de Recherche pour le Développement (IRD), CNRS, Ifremer, LEMAR, IUEM, F-29280 Plouzane, France; moustapha.nouribrahim@univ-brest.fr (M.N.); alain.guenneguez@ird.fr (A.G.); sylvain.petek@ird.fr (S.P.); 2 Centre d’Études et de Recherche de Djibouti, Institut des Sciences de la Vie ISV, Route de l’Aéroport, Haramous BP 486, Djibouti; aafarhan@hotmail.fr (A.A.); abd_daher@yahoo.fr (A.D.); 3 Univ Brest, Institut National de Recherche pour l’Agriculture, l’Alimentation et l’Environnement (INRAE), Laboratoire Universitaire de Biodiversité et Écologie Microbienne, F-29280 Plouzane, France; laurence.meslet@univ-brest.fr; 4 Univ Brest, Plateforme Résonance Magnétique Nucléaire-Résonance Paramagnétique Electronique (RMN-RPE), F-29238 Brest, France; stephane.cerantola@univ-brest.fr (S.C.); gaelle.simon@univ-brest.fr (G.S.)

**Keywords:** antioxidants, eco-friendly extraction, phenolic compounds, LC-MS, NMR, cosmeceuticals, skin aging

## Abstract

This study focuses on developing innovative and eco-friendly purification methods for the isolation of bioactive compounds derived from *Padina pavonica*, a brown abundant macroalga in Djibouti. Three distinct fractions, obtained via liquid-liquid extraction (LLE_FAE), solid-phase extraction (SPE_WE50), and flash chromatography (FC_EtOH20), were selected based on their high phenolic content and antioxidant activities. All fractions were also evaluated for their anti-ageing potential by assessing their ability to inhibit two vital skin-ageing enzymes, tyrosinase and elastase. Structural analysis by ^1^H-^13^C HMBC NMR and LC-MS revealed a selectivity of phlorotannins depending on the purification methods. The LLE_FAE fraction exhibited greater structural complexity, including compounds such as phloroglucinol, diphlorethol/difucol, fucophlorethol and bifuhalol, which likely contribute to its enhanced bioactivity compared to the fractions obtained by FC_EtOH20 and SPE_WE50, which were also active and enriched only in phloroglucinol and fucophlorethol. These findings highlight the impact of purification techniques on the selective enrichment of specific bioactive compounds and demonstrated the interest of FC or SPE in producing active phlorotannin-enriched fractions. These two purification methods hold strong potential for innovative cosmeceutical applications. Results are discussed regarding the use of *P. pavonica* as a promising marine resource in Djibouti to be used for the development of cosmetic industry.

## 1. Introduction

Ultraviolet (UV) radiation exposure is a leading cause of premature skin ageing and various dermatological disorders. UV rays, particularly UVB and UVA, penetrate the skin layers and trigger the excessive generation of free radicals, known as reactive oxygen species (ROS) [1]. These ROS induce oxidative damage to skin cells, leading to the degradation of collagen and elastin, which manifests as wrinkles, loss of elasticity, and pigmentation spots. Furthermore, chronic UV exposure is associated with an increased risk of skin cancers. Developing practical solutions to protect the skin from such environmental aggressions is crucial in light of these harmful effects [2]. While synthetic sunscreens and antioxidant products are commonly used to mitigate the effects of UV radiation, the cosmetic industry is increasingly seeking natural alternatives. Marine-derived compounds with photoprotective and anti-ageing properties offer a promising avenue to meet the growing demand for safer and more sustainable cosmetic solutions [3,4,5].

Polyphenols show promise as photoprotective agents, especially those extracted from marine organisms [6,7,8,9,10,11], due to their ability to absorb UV rays and their antioxidant properties, which enable them to neutralize the reactive oxygen species (ROS) produced by these rays. Among marine organisms, macroalgae, when exposed to unfavorable environmental conditions, produce phenolic compounds to protect themselves against UV radiation and environmental stress [12]. In particular brown seaweeds, which produced phlorotannins, polymers of phloroglucinol units [13,14,15,16,17,18,19,20]. Phlorotannins, classified according to the linkages between phloroglucinol units, are divided into four subclasses, i.e., fuhalols and phlorethols with ether linkages, fucols with phenyl linkages, fucophlorethols with ether and phenyl linkages, and eckols/carmalols with dibenzodioxin linkages, and have a wide range of functional and biological properties, including antioxidant, photoprotective, tyrosinase inhibitory, anti-melanogenic, MMP inhibitory and anti-ageing activities [5,8,17,20,21,22,23,24,25,26].

To sustainably extract phlorotannins, various green methodologies and alternative solvents have been investigated to isolate bioactive compounds while adhering to environmentally responsible practices. Chemat et al. [27] describe green extraction as a process aimed at reducing energy consumption, promoting alternative and renewable solvents, and ensuring safe and high-quality extracts. They also introduced six core principles of green extraction, which should guide researchers and industries in developing innovative and environmentally friendly extraction methods. Prominent approaches include techniques, such as supercritical fluid extraction (SFE), microwave-assisted extraction, enzymatic extraction, ultrasound-assisted extraction, and accelerated solvent extraction (ASE) [24,28,29]. These innovative techniques, such as ASE, are particularly effective in optimizing the extraction and purification of phlorotannins while preserving their structure and bioactive properties [30]. They address critical challenges of green chemistry, particularly in marine algae, by improving extraction yields and preventing the degradation of sensitive compounds [31,32]. Adopting such methods contributes to the sustainability of extraction processes, aligning with the principles of green chemistry and facilitating a more eco-responsible/sustainable valorization of marine resources.

Solid-phase extraction (SPE) and flash chromatography (FC) have been widely used to purify phlorotannins. It was demonstrated in the invasive brown macroalga *Sargassum muticum* with SPE useful to generate sufficiently purified fractions to identify the structure of phlorotannins by 2D NMR [33]. In another work, Gager et al. [30] demonstrated that SPE significantly enhanced the concentration of active fractions compared to the crude extract. Similarly, Buscaglia et al. [34] successfully purified bioactive phlorotannins using flash chromatography, highlighting the effectiveness of this other method for isolating specific bioactive compounds.

The identification and characterization of phlorotannins remain complex due to the lack of reference standards and the intricacy of their polymer structures. To overcome these challenges, advanced analytical techniques are commonly employed. Ultra-high performance liquid chromatography (UHPLC), coupled with quadrupole time-of-flight mass spectrometry (Q-TOF MS/MS), alongside nuclear magnetic resonance (NMR) spectroscopy (^1^H and ^13^C), are among the most effective tools used to accurately identify and characterize the complex molecular profiles of phlorotannins [20,35]. These approaches enable a more precise understanding of their structural diversity and bioactivity, paving the way for their use in cosmetic and health applications.

In this present study, we were interested by an abundant brown macroalga, *Padina pavonica* (Dictyotales, Dictyotaceae), which is found in warm temperate to tropical climates worldwide and is widely available in the waters of the Red Sea [36,37,38]. This brown seaweed is colonizing rockyshores of Djibouti. Nour [39] highlighted its potential for human food, particularly its potent antioxidant activity and richness in phenolic compounds, carotenoids, and minerals, and interestingly, its uses to prepare tartare and wheat for the conception of donuts. *Padina pavonica* is widely recognized for its multiple benefits, including antioxidant, antimicrobial, antibiotic, hypoallergenic, hepatoprotective, anti-inflammatory and anti-diabetic activities [37,40,41,42,43,44]. These characteristics make it a subject of interest in various fields, such as medicine, nutraceutical and cosmetic research. However, the molecular mechanisms underlying these bioactive properties remain largely unexplored. Crude extracts of phenolic compounds showed interesting activities, but the compounds were not structurally identified. This is why *P. pavonica* has been chosen here as a model species for the recovery of active phlorotannins, using eco-friendly extraction and purification methods.

Previous studies have elucidated the structure of phlorotannins in *P. tetrastromatica*, a species within the same genus as our study organism, among these compounds are phloroglucinol and fucophlorethols (tri-, tetra-, penta- and hexamer) [35,45].

In this context, the present study focuses on the use of environmentally friendly extraction and purification methods, including accelerated solvent extraction (ASE), solid-phase extraction (SPE), and flash chromatography (FC), in comparison to the traditional non-friendly liquid-liquid extraction (LLE) method, to isolate for the first time phlorotannin-rich fractions from *P. pavonica*. These purified fractions were subsequently evaluated for their potential cosmetic applications, particularly their anti-ageing properties, by targeting the inhibition of two key enzymes, elastase and tyrosinase, which are central to the degradation of the skin’s structural proteins and in skin ageing. This study aims to provide new insights into the cosmeceutical potential of *P. pavonica*, addressing the growing demand for natural and sustainable ingredients in cosmetic formulations, and it describes for the first time phlorotannins produced by the brown macroalga *P. pavonica*, depending on the purification method. The findings may contribute to innovation in the cosmetic field and enhance the value of local marine resources in Djibouti.

## 2. Results

In this section, we present the crude extract obtained using solvent-accelerated extraction (ASE) from *Padina pavonica*, which was subsequently purified using two eco-friendly methods: solid-phase extraction (SPE) and flash chromatography (FC), compared with the conventional liquid-liquid extraction (LLE) method. The various fractions generated by each method, were evaluated for their phenolic content and antioxidant activities. Only the most active fractions from each method were analyzed using NMR and LC-MS, for which the chemical structure of active phlorotannins has been identified. Finally, these active extracts were tested for their enzymatic inhibition properties to assess their potential for cosmetic applications.

### 2.1. Yield, Phenolic Content and Antioxidant Activity of ASE Crude Extract and Purified Fractions from LLE, SPE, FC Purifications

Table 1 presents the mass yields from ASE, along with the total phenolic content and antioxidant activities of both the crude extracts and the purified fractions obtained via liquid-liquid extraction (LLE_FAE), solid-phase extraction (SPE), and flash chromatography (FC). The crude extract obtained by ASE had a mass yield of 18.2%. The total phenolic content of the crude extract was 94.9 mg PGE/g DW. Liquid-liquid purification (LLE_FAE) was carried out, resulting in a fraction containing 155.1 mg PGE/g DW, which was more than 1.6 times more concentrated than the crude extract. DPPH IC50 radical scavenging and FRAP EC50 antioxidant activities were measured on the crude and purified extract (Table 1). After purification, a decrease in IC50 and EC50 was recorded for some fractions, reflecting an increase in activities due to the concentration of phlorotannins, particularly in three fractions, one for each purification method, which corresponded to LLE_FAE, FC_EtOH20 and SPE_WE50, fractions.

The SPE purification procedure was carried out by elution from four solvents of decreasing polarity: Water (W), Water-Ethanol (1:1) (WE50), Ethanol (E), Ethanol-Dichloromethane (1:1) (ED) and DCM (D). For flash chromatography (FC), the various peaks were collected between water and ethanol. The EtOH20 FC fraction had the highest TPC of the other fractions, with a phenolic content of 132.1 mg/g DW (Kruskal-Wallis, *p* < 0.0001), 1.39 times higher than the activity of the crude ASE extract. The SPE procedure concentrated phenolic content on the WE50 fraction with 129.4 mg/g DW (Kruskal-Wallis, *p* < 0.001), indicating that the phenolic content was multiplied by 1.36.

According to the results of the DPPH radical scavenging activity assay, and among eco-friendly methods, the FC_EtOH20 fraction exhibited the highest activity, closely matching the positive control (0.02 and 0.01 for respectively Vitamin C and BHA), compared to the other FC fractions, with an IC50 value significantly lower (0.06 mg/mL) than that of the crude ASE extract (0.29 mg/mL) (Table 1). A significant difference was observed between all the FC fractions generated by the process (Kruskal-Wallis, *p* < 0.001). Regarding the SPE method, the WE50 and E fractions exhibited the most potent free radical scavenging activities (respectively 0.10 and 0.16 mg/mL, approaching the positive controls), compared to the other SPE fractions, with the SPE_WE50 significantly more interesting with an IC50 of 0.10 mg/mL than the other fractions generated by the process (Table 1).

In terms of antioxidant activity measured by the FRAP assay, the EtOH20 fraction from the FC method displayed remarkable activity, with an EC50 of 0.12 mg/mL, closely approaching that of the positive controls (respectively for vitamin C and BHA, 0.08 and 0.09 mg/mL), and outperforming both the crude ASE extract (0.25 mg/mL) and other FC fractions. For the SPE method, the WE50 fraction exhibited an interesting EC50 value (0.15 mg/mL), followed by the E fraction with 0.22 mg/mL, which were closer to the positive controls than the crude ASE extract and other SPE fractions. No significant differences were observed (*p* = 0.08 and *p* = 0.10, respectively).

The most active fraction remains the fraction LLE_FAE generated by the liquid partition washing protocol, which is not eco-friendly, and which generated an active fraction with an IC50 of 0.03 mg/mL and an EC50 of 0.11 mg/mL, very close to the values obtained for the positive controls, vitamin C (0.02 and 0.08 mg/mL) and BHA (0.01 and 0.09 mg/mL).

### 2.2. Enzymatic Activity of ASE Crude Extract and Purified Fractions from LLE, SPE, and FC Methods

**Figure 1:**
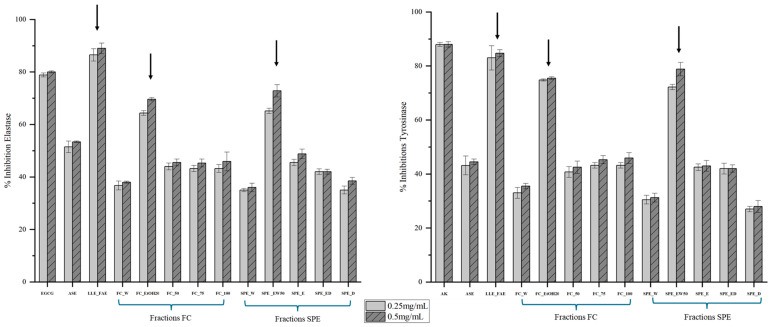
Anti-ageing activities by elastase and tyrosinase inhibitions, determined on the different fractions, LLE_FAE, SPE and FC and their crude respective ASE extracts, obtained from the brown macroalga *Padina pavonica*. Epigallocatechin gallate (EGCG) and kojic acid are positive controls. Purified fractions which are used in our comparative study are shown by the arrows.

The inhibition percentages of elastase and tyrosinase at two distinct concentrations, 0.25 and 0.5 mg/mL, for the FC, SPE, and LLE_FAE fractions, as well as the crude extract, are presented in Figure 1. The 0.5 mg/mL concentration exhibited the most significant inhibitory effects, so subsequent results focus on this concentration. The anti-elastase activity tests revealed significant differences across all extracts and fractions (Kruskal-Wallis, *p* = 0.02). The ethyl acetate fraction from liquid-liquid extraction (LLE_FAE) demonstrated the highest inhibition percentage at 89.0 ± 1.26%, slightly surpassing the positive control, epigallocatechin, which achieved 79.6 ± 0.01% (Figure 1). This was followed by the SPE_WE50 fraction, which showed 72.9 ± 4.3% inhibition, marginally higher than the FC_EtOH20 fraction purified by flash chromatography, with 69.6 ± 1.6%. The remaining fractions exhibited less than 50% inhibition, indicating lower enzymatic inhibitory activities. The moderately pronounced activity was recorded in the crude extract with an average of 52.3 ± 0.51%.

Regarding tyrosinase inhibition, the percentages of enzyme inhibition for the various fractions and extracts, along with kojic acid as a positive control, are shown in Figure 1. The results were significantly different (Kruskal-Wallis, *p* = 0.02). The LLE_FAE fraction exhibited the highest inhibition percentage at 84.5 ± 1.53%, closely approaching that of the positive control, kojic acid, which showed 88.96 ± 1.05%. Among the SPE and FC fractions, only SPE_WE50 and FC_EtOH20 demonstrated significant inhibitory activities, with inhibition rates of 78.8 ± 1.75% and 75.5 ± 2.06%, respectively. The remaining fractions, as well as the crude extract, did not exhibit notable inhibition.

Further analyses using NMR and LC-MS will focus on these active fractions, to identify compounds responsible of the activities.

### 2.3. ^1^H NMR and ^13^C NMR (HMBC) Identifications of Phlorotannins-Rich Fractions from SPE (WE50), Flash Chromatography (FC_EtOH20) and Liquid Partitions (LLE_FAE)

**Figure 2:**
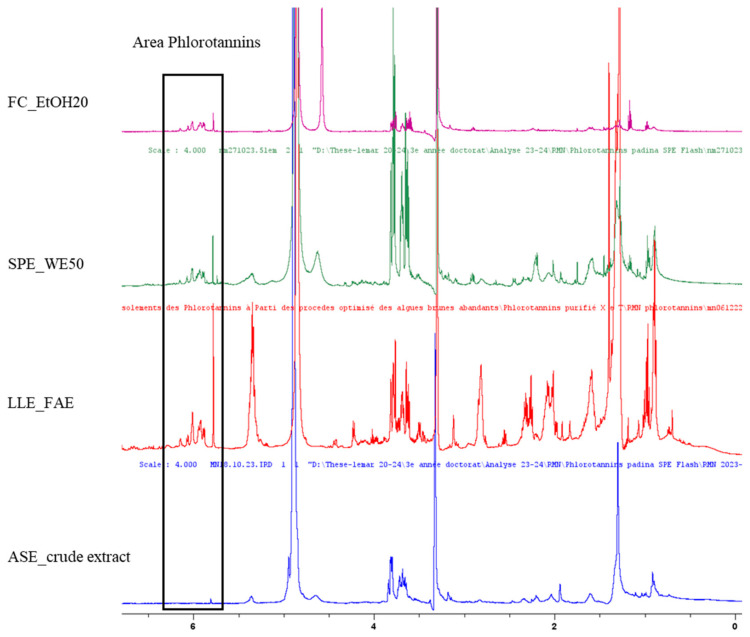
^1^H NMR spectra on phlorotannin-rich fractions from the brown macroalga *Padina pavonica* and obtained by three separate purification methods and the name of fractions into parentheses: solid phase extraction (SPE_EW50), flash-chromatography (FC_EtOH20), purification by washing and noted liquid/liquid (LLE_FAE) and comparison with the spectrum from the crude ASE extract (Accelerated Solvent Extraction).

The ^1^H NMR spectrum of the LLE_FAE, SPE_WE50 and Flash chromatography FC_EtOH20 fractions of the samples, in comparison with the crude ASE extract, showed signals corresponding to phenolic compounds located between 5.5 and 6.5 ppm. The ^1^H NMR spectrum of the crude ASE extract of *P. pavonica* (Crude extract; Figure 2) showed mainly signals for carbohydrates, in particular mannitol between 3.6 and 4.0 ppm, as well as 0.5 and 2.8 ppm, which can be attributed to amino acids and apolar compounds, such as fatty acids. The mannitol signals are high for the LLE and SPE techniques, while they are weak for the other FC purification technique. Moreover, ^1^H NMR analysis of the ethyl acetate fraction (LLE_FAE) revealed the presence of a singular signal at 5.98 ppm, more elevated for LLE and less elevated for SPE and FC and which corresponds to phloroglucinol, together with overlapping signals between 5.9 and 6.2 ppm, typical of phlorotannins, accompanied by a decrease in peaks associated with mannitol (Figure 2). The WE50 fraction from the SPE procedure also showed phlorotannin signals between 5.98 and 6.2 ppm, although less pronounced than those of the FAE fraction, with a significant presence of mannitol and fewer signals from apolar compounds. On the other hand, the FC_EtOH20 fraction revealed less phlorotannin signals compared to the SPE_WE50 fraction and with a big reduction in mannitol and lipid compound signals (Figure 2).

The two dimensional ^1^H-^13^C NMR analyses, combining data from ^1^H NMR and ^13^C NMR analyses (Figure S1, Supplementary Materials), proved the presence of aromatic signals, then polyphenolic compounds in all three purified fractions of *P. pavonica* (Table 2). Indeed, signals near δ = 92 ppm were detected in all three purified samples, identified as a methine carbon (C-H of the aromatic rings). In addition, carbon peaks at δ = 160 ppm were identified as phenolic carbons (Table 2). Also noteworthy is the presence of a common signal around δ = 125–130 ppm, consistent with a diaryl ether bond, suggesting the presence of linear phlorethol-type polymer/s, and also signals near δ = 100 ppm corresponding to carbons involved in an aryl-aryl bond between two PG units, and suggesting the presence of fucol-type polymer/s. According to these NMR data and combining the 2D NMR obtained for the three fractions, different compounds could be identified, as the monomer phloroglucinol as suggested in the brown Sargassaceae macroalga *Ericaria selaginoides* by Jégou et al. [46], fucol, phlorethol and also a fucophlorethol. Phloroglucinol and a fucophlorethol are present in the three fractions LLE_FAE, SPE_WE50 and FC_EtOH20, as presented in the Table 2. Observed only in the LLE_FAE, another carbon peak appearing between δ = 145 and 150 ppm indicating the presence of a fuhalol-type phlorotannin.

These NMR data were confirmed by mass spectrometry analysis, which reveals the presence of four different phlorotannins in the fraction LLE_FAE, i.e., phloroglucinol, phlorethol or fucol (dimer), fucophlorethol (tetramer) and fuhalol (dimer), and only two phlorotannins in the SPE_WE50 and FC_EtOH20 purified fractions, phloroglucinol and fucophlorethol (tetramer) (Table 3).

Interestingly, our work shows that depending on the purification procedure, detected phlorotannins are not the same when comparing both purification procedures, i.e., liquid partitions versus eco-friendly methods. The classical procedure using washings, LLE_FAE, permitted to obtain all the structures of phlorotannins, the monomer phloroglucinol, phlorethol/fucol, fucophlorethol and fuhalol (Table 2 and Table 3), while in both others eco-friendly procedures, the fuhalol structure and dimers were not detected.

### 2.4. LC-ESI-QTOF-MS Analysis of Phlorotannins

LC-MS analysis of the LLE_FAE, SPE_WE50, and FC_EtOH20 fractions of *P. pavonica* showed the presence of various phlorotannins with a range of [M − H]^−^ *m*/*z* from 125.0255 to 374.2470 and retention times (RT) from 0.85 to 1.11 (Table 3). Chromatograms and mass spectra of elucidated phlorotannins are presented as Supplementary Materials (Figure S2 and Figure S3, respectively). Due to the lack of commercially available standards for phlorotannin oligomers, we based the identification of *P. pavonica*’s phlorotannins on the MS spectral data together with published literature. Our findings are consistent with the phlorotannin structures reported in previous studies carried on brown macroalgae, especially on *P. tetrastromatica* for the two first papers [35,45,48].

One should note that phlorotannins produced by *P. pavonica* present a molecular weight (MW) between 126 to 375 Da (Table 3). Then, phlorotannins produced by the Djiboutian species are not very polymerized: only a monomer, dimers and trimers have been isolated and elucidated and constituted the pool of phlorotannins produced by the brown macroalga *P. pavonica* from Djibouti.

### 2.5. LC-ESI-QTOF-MS Analysis of Others Compounds

The three purified fractions, LLE_FAE, FC_EtOH20 and SPE_WE50, are also enriched in other compounds as listed in Table 4. Many compounds are currently being elucidated, and we proposed some names of compounds (Table 4).

The SPE_WE50 fraction has the greatest diversity of compounds with seven listed, followed by the LLE_FAE and FC_EtOH20 fractions with six compounds (Table 4).

Some lipidic compounds were co-extracted from *P. pavonica* and consisted in fatty acids (arachidonic acid in LLE_FAE, phtalonic acid in FC_EtOH20 and SPE_WE50), terpenes (dolastanes and dictyotadiol in LLE_FAE, (+)-epiloliolide in FC_EtOH20), and oxylipins (cymatherol B in FC_EtOH20) (Table 4). Additionally, a flavone (licoflavone C) and some carbohydrates were also present in the purified fractions: mannitol isolated in the three fractions, a glucoside in both eco-friendly fractions and 4-[(E)-2-Carboxyvinyl]-2-methoxyphenyl β-D-glucopyranosiduronic acid isolated in the SPE fraction.

It is interesting to note that the carbohydrate mannitol was consistently present across all three purification approaches, indicating its broad solubility and availability in the extracts regardless of the method applied. Arachidonic acid/dictyotadiol, dolastanes, licoflavone C/(+)-epiloliolide and compound 12 are present only in the LLE_FAE fraction, while penicitide B/carnosol and compound 10 are present only in the SPE_WE50 fraction. The molecule Hexyl 2F-furoate was explicitly identified within the FC_EtOH20 fraction. Interestingly, some other compounds were isolated only in the two eco-friendly procedures: cymatherol B, phtalonic acid and Peonidin 3-O-sambubioside-5-O-glucoside were found only in SPE_WE50 and FC_EtOH20 fractions, demonstrating method overlap in compound extraction.

Some surprising compounds were also detected, as asperterphenyl (in LLE_FAE), asperbiphenyl (in the three fractions), penicitide B (in SPE_WE50), which were isolated and identified from fungi (MarinLit data).

Interesting is the compound 12 (Table 4), present only in the LLE_FAE fraction, which could be affiliated to the 4′-[3-Hydroxy-5-(2,4,6-trihydroxyphenoxy)phenoxy]-2,3′,4,5′,6-biphenylpentol, a fucophlorethol (tetramer) phlorotannin by its mass (482 Da) and visible in the literature. Nevertheless, its particular ionisation (Figure S4), and particularly the M + 2 signal higher than the M + 1, leads rather to a chlorinated compound. The compound 12, with a potential and proposed (chemical structure of C_24_H_47_ClO_7_ (absent from MarinLit and ChemSpider data bases), is currently being elucidated.

## 3. Discussion

### 3.1. Interest to ASE to Concentrate Phenolic Compounds

Accelerated solvent extraction (ASE) has demonstrated significant yield, producing 18% crude extract of the brow macroalga *Padina pavonica* studied in the present study. Previous studies, such as those conducted by Heffernan et al. [49] and Gager et al. [30], working on other brown seaweeds, have reported higher extraction yields using the ASE method on various brown seaweeds, specifically yields of 31.7% were obtained from *Fucus serratus*, 24.8% from *Laminaria digitata*, 53.9% from *Halidrys siliquosa*, and 27.6% from *Ascophyllum nodosum*. This increased efficiency attributed to ASE being used at elevated temperatures and pressures, enabling better extraction of the desired compounds as suggested by Tanniou et al. [32], without any degradation of compounds. In our study, *P. pavonica* was found to have a TPC content of 94.9 mg PGE/g extract when subjected to assisted pressure extraction (ASE). Similar results were obtained for *Ascophyllum nodosum* using pressure-assisted extraction (ASE), with a 101.30 mg PGE/g reported by Tanniou et al. [32]. However, *Padina* sp. showed slightly higher results using the ultrasound-assisted extraction (UAE) method, with a value of 124.65 mg GAE/g [50], on the other hand, maceration resulted in significantly lower TPC content on *P. pavonica* with a value of 7.06 mg PGE/g, as suggested by Abdelhamid et al. [51].

Several studies have shown significant results for extracting phenolic compounds from ASE algae [30,46,52]. According to our present study, *P. pavonica*’s crude extract obtained through ASE procedure has a scavenging activity (IC50) of 0.29 mg/mL. However, *P. pavonica* extracted using the solid/liquid (S/L) method demonstrated a threefold lower free radical scavenging activity, with a concentration of 0.91 mg/mL, as reported by Abdelhamid et al. [51], with similar findings suggesting that ASE extracts permitted the obtaining of most active compounds [32].

### 3.2. Comparison of the Purification Procedures to Obtain Active Phenolic Compounds

The results of this study highlighted the effectiveness of various extraction and purification methods in enriching the phenolic compounds from *P. pavonica*, in line with the principles of green chemistry. The two eco-friendly methods, solid-phase extraction (SPE) and flash chromatography (FC), as well as the comparative liquid-liquid extraction (LLE), significantly concentrated the phlorotannins compared to the crude extract obtained through accelerated solvent extraction (ASE) as suggested by Montero et al. [28] and Gager et al. [30]. The results of this study demonstrated that the liquid-liquid purification method successfully yielded an ethyl acetate fraction (LLE_FAE) with a total phenolic content (TPC) of 155.1 mg/g DW, indicating a significant concentration of phenolic compounds. These findings are comparable to those reported by Hassan et al. [53] and Naw et al. [54], who observed even higher phenolic content in the ethyl acetate fractions of *Padina australis* (807.20 mg/g DW) and *P. tetrastromatica* (589.79 mg/g DW). Ethyl acetate has proven to be an effective solvent for the selective extraction of polyphenolic compounds, as demonstrated in various studies on algae [8,22,55]. Similarly, the EtOH20 fraction obtained through flash chromatography (FC) also exhibited high levels of TPC. Comparable findings were reported by Muñoz-Quintana et al. [56], who demonstrated that the EtOH20 fraction obtained through FC contained approximately 829.6 mg/g DW, which is 2.5 times higher than the crude extract obtained through ASE from *Halidrys siliquosa*. Gager et al. [30] also utilized SPE to enrich the phenolic content of the ASE crude extract from *Halidrys siliquosa*, increasing the phenolic concentration from 190.5 mg/g DW to 395.5 mg/g DW in the water:ethanol fraction (1:1). Similarly, Zubia et al. [29] demonstrated the effectiveness of SPE in concentrating phenolic compounds from the crude extract of *Bifurcaria bifurcata*, increasing from 1% dry weight to 13.3% dry weight in the aqueous SPE fraction. Additionally, Rajauria et al. [57] reported a 1.6-fold increase in phenolic content when ethanol was used to enrich the crude extract of *Himanthalia elongata* (S/L), with levels rising from 178.2 mg/g DW to 279.2 mg/g DW in the hydromethanolic fraction. This study demonstrates significant differences in the total phenolic content (TPC) levels between the ASE crude extract and the fractions purified by LLE, SPE, and FC (Kruskal-Wallis, *p* < 0.001), showing that the purification processes substantially influence the concentration of bioactive compounds.

Regarding antioxidant activities, the purification methods had a significant impact, as demonstrated by our results, with the traditional purification procedure, LLE, giving the best activities. Sharma and John [35] reported that the ethyl acetate fraction of *P. tetrastomastica* exhibited notable antioxidant activity, which aligns with our observations for the LLE_FAE fraction obtained in this study. The low IC50 value observed for the LLE_FAE fraction (0.03 mg/mL) could be attributed to the phenolic richness of this fraction, explaining its DPPH radical scavenging activity, as also suggested by Chia et al. [58]. Interestingly, the EtOH20 fraction obtained through Flash Chromatography (FC) displayed the most promising antioxidant activity, with an IC50 lower than that of the other FC fractions, reaching 0.06 mg/mL. This result correlates with the high levels of phenolic compounds found in this fraction, which could explain its potent antioxidant activity, as suggested by Muñoz-Quintana et al. [56] in a similar study. In the case of Solid Phase Extraction (SPE), the SPE_WE50 and E fractions showed the best radical scavenging activities, with IC50 values of 0.10 mg/mL and 0.16 mg/mL, respectively. In the literature, Onofrejová et al. [59] have found that antioxidant activities were similar between ASE extract and SPE fractions for the Laminariales *Undaria pinnatifida*. Results are consistent with those reported for the H2O-EtOH and EtOH fractions of SPE for *Halidrys siliquosa*, which had IC50 values of 0.10 mg/mL and 0.11 mg/mL, respectively [30]. These findings suggest that the choice of purification method plays a critical role in concentrating bioactive compounds, particularly phlorotannins responsible for antioxidant activity. The marked improvement in DPPH radical scavenging capacity in certain purified fractions highlights the effectiveness of these methods in enriching fractions with phenolic compounds exhibiting potent antioxidant properties. FRAP activity, which measures the ability to reduce ferric ions to ferrous ions, is an important indicator of antioxidant capacity [60]. The LLE_FAE, FC_EtOH20, and SPE_WE50 fractions showed notable FRAP antioxidant activities, highlighting their potential in reducing oxidative stress. Similar results were observed with the FAE fraction of *P. tetrastromatica*, which exhibited a FRAP value of 0.26 mg/mL [35]. However, as seen with the DPPH assay, the purification method influences the selectivity of bioactive compounds. This suggests that different techniques may selectively concentrate phenolic compounds responsible for specific antioxidant mechanisms.

### 3.3. Anti-Ageing Activities in Fractions Rich in Plorotannins

Inhibition of the enzyme elastase, which degrades elastin in the skin’s extracellular matrix, can protect against photodamage and other structural damage. This improves skin elasticity and reduces visible signs of ageing, such as dryness, wrinkles, and volume loss [1]. The effects of marine algae compounds have recently garnered attention in anti-ageing research over the decade [61,62]. The results of the anti-elastase inhibitory effects of various fractions from FC, SPE, and the comparative LLE method are presented in Figure 1. Only the three fractions that were rich in phenolic compounds and exhibited strong antioxidant activities also showed significant enzymatic inhibition against elastase and tyrosinase. Among them, the LLE_FAE fraction demonstrated the highest inhibitory effect on elastase, reaching 89%, slightly surpassing the inhibition by EGCG (positive control), which was 79.9%. This result was notably higher compared to the FC_EtOH20 and SPE_WE50 fractions. Similarly, the LLE_FAE fraction from *P. tetrastromatica* also displayed a significant anti-elastase activity, outperforming the positive control EGCG, as reported by Sharma and John [35]. The SPE-WE50 and FC-EtOH20 fractions showed significant inhibition rates of 72.9% and 69.1%, respectively. Gager et al. [30] and Jesumani et al. [63] described that the phlorotannin-rich fractions *Ascophyllum nodosum* and *Sargassum vachellianum* showed significant anti-elastase activity reaching 70.6%. In a recent study, significant inhibition of the elastase enzyme was observed with ‘Fraction buthanol’ (F-BuOH) from *Sargassum horridum*, reaching a level of 30.89%, which was also the most antioxidant extract [64]. In our study, these three fractions showed significant antioxidant activities correlated to the different compounds identified in these fractions (Table 2 and Table 3, see Section 2.3, Section 2.4 and Section 2.5). Tyrosinase catalyzes the conversion of tyrosine to dopaquinone, which is then auto-oxidized to L-DOPA and L-dopachrome, and ultimately converted to melanin [22]. Sun exposure increases tyrosinase and melanosome production, leading to hyperpigmentation; simultaneously, consumers are shifting away from chemical ingredients towards natural and environmentally sustainable cosmetic products, with compounds derived from marine algae, including microalgae biomass extracts, gaining popularity for their cosmeceutical applications [65]. Like elastase inhibition, the three phlorotannin-rich fractions exhibit significant results for tyrosinase inhibition, with LLE_FAE > SPE_WE50 = FC_EtOH20 in order of potency. The strong tyrosinase and elastase inhibition observed in the LLE_FAE fraction correlates with its high concentration of phenolic compounds. Regarding our interesting results, these extracts, particularly SPE_WE50, could be used as active ingredients in the cosmetics or even cosmeceutical sector, to combat the effects of time on skin elasticity and pigmentation.

### 3.4. Structural Elucidation of Phlorotannins Using NMR and LC-ESI-QTOF-MS Techniques

**Figure 3:**
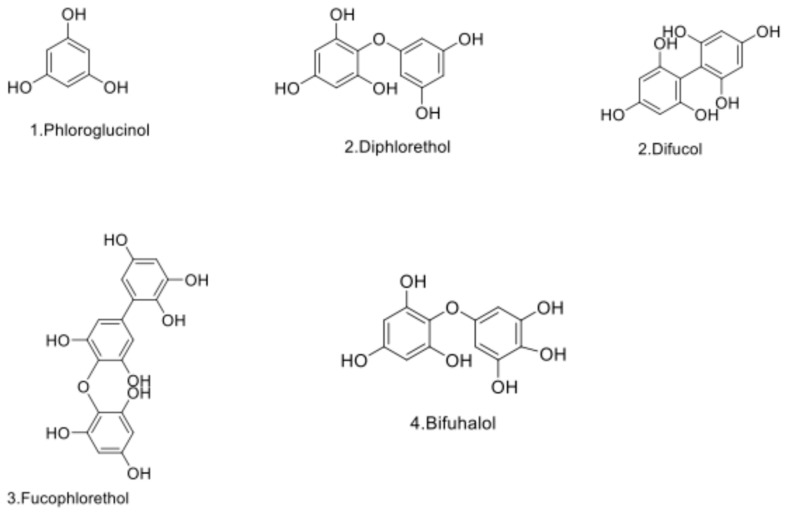
Structures of phenolic compounds identified in the brown macroalga *Padina pavonica* and referenced and numbered like in Table 3.

The identification and the structural elucidation of phenolic compounds in this study were carried out using advanced analytical techniques. Two-dimensional Nuclear Magnetic Resonance (2D NMR) spectroscopy was employed alongside Liquid Chromatography-Electrospray Ionization-Quadrupole Time of Flight-Mass Spectrometry (LC-ESI-QTOF-MS/MS) for detailed molecular identification [55,66]. Kergosien et al. [67] utilized ^1^H NMR and 2D NMR to successfully identify the structure of phlorotannins in species from the *Sargassum* genus. These analyses were conducted on the two eco-friendly fractions, as well as on the comparative LLE_FAE fraction, all enriched in phlorotannins. This approach also enabled the characterization of additional compounds present in the purified fractions. Due to the lack of commercially available standards for phlorotannin oligomers, we followed published literature to identify phlorotannins in the *Padina* genus. Our findings align with the phlorotannin structures reported in previous studies [35,45,48]. This highlights the effectiveness of these techniques in characterizing complex phenolic structures, further supporting their application in our study for the elucidation of phlorotannins in *P. pavonica*. LC-MS analysis indicated the presence of a monomeric molecule with a monoisotopic mass of [M + H]^+^ at 127.039 *m*/*z* and [M − H]^−^ at 125.023 (RT = 0.5 min) specifically within the LL_FAE fraction. Additionally, this compound appeared as a singlet in the ^1^H NMR spectrum for this fraction, with a weaker signal observed in the two other fractions (see Figure 2). This molecule has been identified as phloroglucinol, a phlorotannin monomer, as already demonstrated in *P. tetrastromatica* [45]. Another molecule, with a monoisotopic mass of [M + H] at 251.053 *m*/*z*, was identified in this fraction. Based on the observed mass spectrometric fragmentation profile and comparison with published literature [45,55], this phlorotannin has been tentatively identified as either diphlorethol or difucol. During the eco-friendly purification of fractions, a molecule with a monoisotopic mass of *m*/*z* 374.2522 was identified in negative mode, and it was also detected in the LLE_FAE fraction. Additionally, the HMBC spectrum revealed a characteristic diaryl-ether carbon linkage between 125 and 130 ppm, supporting the identification of this phlorotannin trimer as fucophlorethol. A phlorotannin with similar precursor and product ions was previously studied in detail from the brown macroalga *Cystoseira usneoides* (Fucales), which also contained three phloroglucinol units [68]. Based on comparisons with published data [45,55,69,70], this phlorotannin has been identified as fucophlorethol composed of three units of phloroglucinol (trimer). Figure 3 presents chemical structures of phlorotannins isolated from the brown macroalga *P. pavonica* from Djibouti.

In our study, the selectivity of phlorotannins derived from eco-friendly and conventional purification methods was highlighted. One should note that phlorotannins produced by *P. pavonica* presents a weak degree of polymerization (DP3 here), compared to others big phlorotannins, isolated from brown macroalgae, like DP13 found in some temperate Fucales [20].

These findings suggest that purification methods exhibit selectivity concerning the nature of phenolic compounds, but also of others compounds, as proposed by Gager et al. [30]. Furthermore, these observations support the notion that fractions rich in phenolic compounds are linearly correlated with antioxidant and enzymatic activities. Notably, the LLE-FAE fraction demonstrated a higher yield and phenolic content compared to the two eco-friendly fractions, SPE_WE50 and FC_EtOH20. This richness in phenolic compounds in the LLE-FAE fraction could account for its biological activity, including its efficacy in anti-ageing and antioxidant properties, as suggested by previous research [64,71]. Nevertheless, the eco-friendly fractions, regardless of their concentrations of phlorotannins, exhibited interesting antioxidant activities and cosmetic properties. In order to gain time, solvent and energy, we could then preconize to use regarless FC or SE to isolate phlorotannins instead of the non-friendly procedure LLE. The LLE_FAE permitted to generate a diversified fraction with four different phlorotannins, while FC and SPE procedures concentrated only two phlorotannins. Nevertheless, these FC_EtOH20 and SPE_WE50 were active, which suggests that phloroglucinol and fucophlorethol are the active molecules in the fraction.

Additionally, several other compounds, such as lipids of different categories, carbohydrates and flavone, were identified, with the majority appearing in the LLE-FAE fraction (Table 4).

Interesting was the identification of compounds produced by fungi, and for some, identified in the three different fractions. One should hypothesize the existence of fungal endophytes and/or epiphytes colonizing *Padina* tissues, and at the origine of the production of these fungal molecules (compounds 4, 5, and 9, Table 4).

Our study showed the potential of the brown macroalga *P. pavonica*. This species produces phlorotannins with antioxidant and anti-ageing activities and could be used as a source of ingredients in the formulation of anti-ageing cosmetic products. The use of ethanol and water in the two eco-friendly procedures are enabling the extraction and purification protocols to be scaled up by industrials. Once at the industrial site, an ASE extraction followed by SPE purification would be advisable in order to provide the SPE-WE50 fraction for formulation in the cosmetic sector, and in order to be able to scale up from an industrial point of view.

The question of biomass availability could be raised, given the small size of *P. pavonica* individuals. To develop a potential valorization of the species in Djibouti, it might be worth considering harvesting the species, or even growing it from phycoculture, to obtain large quantities of *P. pavonica*’s active ingredients.

## 4. Materials and Methods

### 4.1. Algal Material

The brown seaweed (Phaeophyceae, Dictyotales, Dictyotaceae) *Padina pavonica* (Linnaeus) Thivy was collected in July 2022 from the Khor Ambado area (southwest of the Gulf of Tadjourah) 11°37.355′ N 43°08.641′ E. Fresh algae samples were carefully washed with tap water and then distilled water to remove sand, shells, and epiphytes. Samples were then stored in a freezer at −20 °C. A voucher sample of *P. pavonica* (AD028) was deposited in the Herbarium collection of the marine biology laboratory at CERD. The specific identification of specimens of *Padina pavonica* was done by the second author (V.S.-P.), by carrying out a morphological analysis of a large number of samples, consisting of transverse sections of the flabellate thallus, comparing the arrangement of the cells, surface view, form and position according to hairlines of sori, with the literature [72,73,74].

### 4.2. Extraction Using Acceleration Solvent Extraction (ASE)

Freeze-dried samples were extracted using an accelerated solvent extraction system (Dionex ASE 150, Sunnyvale, CA, USA). A mixture of 15 g freeze-dried seaweed powder and 30 g Fontainebleau sand was poured into a 100 mL stainless steel extraction cell fitted with glass fiber filters at the ends. A mixture of ethanol:distilled water (70:30) was used as a solvent, static time (the time the solvent remained in the cell) was 5 min (cycle time), and pressure was set at 100 bar (=1500 psi). Extractions were performed with the cell inverted at 40 °C. The rinse volume applied was 100% and the purge time was 120 s. After extraction, the solvent was evaporated using a rotary evaporator (Laborota 4000 efficient, Heidolph, Germany) and frozen at −20 °C before being freeze-dried to form the crude extract, named XE70.

### 4.3. Purifications

Two eco-friendly procedures, i.e., Solid Phase Extraction (SPE) and Flash chromatography, were tested, in comparison with a traditional way to purify compounds using washings using successive solvents of different polarity (Liquid liquid semi-purification).

#### 4.3.1. Liquid-Liquid Semi-Purification of Phlorotannins (LLE)

The ASE extract from *P. pavonica* was used for semi-purification by liquid-liquid extraction. The purification was carried out in 3 separate steps according to the method developed by Stiger-Pouvreau et al. [15], crude extract was subjected to solvent fractionation, the extract was concentrated *under vacuum* to approximately 100 mL, and the residue was split into three times with the same volume of (1) dichloromethane to remove lipids (2) then acetone precipitation followed by ethanol precipitation to remove proteins and carbohydrates, and then (3) ethyl acetate to obtain two fractions, the aqueous fraction and the phenolic-rich ethyl acetate fraction (FAE).

#### 4.3.2. Semi-Purification of Phlorotannins by Solid Phase Extraction (SPE)

The ASE extract from *P. pavonica* was used for semi-purification by solid phase extraction SPE (Vac Elut SPS 24, Agilent Technologies, Santa Clara, CA, USA) on C18 column, (Strata C18-E, 55 μm, 70 A, 20 g/60 mL, Giga Tubes, Phenomenex, Torrance, CA, USA) capable of retaining apolar compounds and allowing polar compounds such as phenolics to pass through. The SPE column was first activated and conditioned with ethanol and water respectively, followed by the deposition of 1 g of dry extract mixed with C18 silica. Different solvents were then added in a polarity gradient from the most polar to the most apolar: distilled water, distilled water: ethanol (50:50, *v*:*v*), absolute ethanol, ethanol: dichloromethane (50:50) and at last dichloromethane. Solid:liquid partitions were used to remove unwanted compounds, such as polysaccharides, proteins eliminated in the first wash with water and pure ethanol, and also lipids eliminated using dichloromethane. The fractions were then evaporated using a rotary evaporator (Laborota 4000 efficient, Heidolph, Schwabach, Germany) and frozen and dried (Beta 1-8 LD, Christ, Hagen, Germany). The TPC was expressed in mg PGE/g of dry fraction extract, which shows the concentration of phenolics in relation to the crude ASE extract.

#### 4.3.3. Flash Chromatography Semi-Purification of Phlorotannins

The ASE extract of *P. pavonica* was fractionated by flash chromatography. The column was chosen according to the polarities of the molecules to be selected, and to achieve a reverse-mode separation (apolar stationary phase), we used a C18 silica reversed phase column (PF-30C18HP-F0025). The column was first activated with 5 column volumes of methanol, then conditioned with the first solvent used, i.e., 5 column volumes of Milli-Q water. A quantity of 1.2 g of crude extract obtained by ASE 300 was solubilized in 1 mL of ethanol and 200 μL of Milli-Q water, then mixed with 3.6 g of C18 silica. The extract incorporated into the silica was then deposited as a solid on the column. Separation was then carried out by elution steps at a flow rate of 15 mL.min^−1^ using different solvents and 4 column volumes per step. The solvents used were Milli-Q water (H_2_O), ethanol (EtOH100) and finally dichloromethane (DCM100), all miscible and used at different ratios. Each fraction was recovered in a bottle, evaporated, taken up in water, frozen and freeze-dried. The TPC was expressed in mg PGE/g of dry fraction extract, which shows the concentration of phenolics in relation to the crude ASE extract.

### 4.4. Spectroscopic Analysis of Phenolic Compounds

#### 4.4.1. NMR Analysis

Molecular signatures of extracts/fractions were obtained by proton (^1^H) Nuclear Magnetic Resonance (NMR) using a Bruker Avance 400 spectrometer from the UBO NMR platform. For each analysis, 5 mg of extract was dissolved in 700 μL of deuterated solvent (either methanol or water). Spectra were analysed using MestReNova software (v 6.0.2-5475). The NMR spectra provide information on the composition of the extracts/fractions, in particular the signal of phenolic compounds appearing between 6.5 and 5.5 ppm.

For 2-D NMR analyses, approximately 10 mg of dry extracts were dissolved in 800 μL of deuterated methanol (CD_3_OD). All spectra were recorded on a Bruker Avance 500 spectrometer using standard pulse sequences available through the Bruker software TopSpin 3.7.0 (Bruker, Paris, France). Chemical shifts were reported in parts per million ppm. The identification of phlorotannins present in the purified fractions was achieved through distortionless enhancement by polarization transfer DEPT, heteronuclear multiple quantum coherence (HMQC), and heteronuclear multiple bond correlation (HMBC) experiments. These were further compared with ^1^H chemical shifts (ranging from 5.5 to 6.5 ppm) and ^13^C shifts (between 95 and 165 ppm) from literature references (Table 1). These signals aid in classifying the phlorotannins based on the bonding patterns.

#### 4.4.2. LC-MS Analysis of Phenolic Fractions

Phlorotannin extracts were resuspended in milliQ water to obtain a 10 mg/mL stock solution. Each extraction was supplemented with three volumes of acetonitrile (containing 0.1% formic acid) and filtered on a 0.45 µm filter. Products were analyzed on Agilent 6530 Accurate-Mass Q-ToF LC-MS (Agilent Technologies, Santa Clara, CA, USA) using an InfinityLab Poroshell 120 HILIC column (2.1 × 100 mm, 2.7 µm) (Agilent technologies). Isocratic elution was performed with 10% solvent A (10 mM ammonium formate buffer containing 0.1% formic acid) and 90% solvent B (H_2_O/acetronitrile + 10 mM ammonium formate buffer + 0.1% formic acid) at 0.250 mL.min^−1^. All samples were analyzed twice for negative (ESI−) and positive (ESI+) modes. Acquired data were analyzed using MassHunter Qualitative Analysis version B.06.00. Identification was done based on comparison between theoretical/experimental *m*/*z*, using the probability given for each chemical formulae proposed by the software, together with literature data.

### 4.5. Total Phenolic Content of Extracts/Fractions

TPC was determined by spectrophotometry using an adapted Folin-Ciocalteu assay [5]. Wells were filled with 20 μL of extract (2 mg/mL), 130 μL of distilled water, and 10 μL of Folin-Ciocalteu reagent followed by 40 μL of Na_2_CO_3_ (200 g/L). The mixture was left for 10 min at 70 °C. The microplate was then placed on ice to stop the chemical reaction, and the absorbance was read at 620 nm. Standard phloroglucinol solutions were also submitted to this assay to obtain a calibration curve. The TPC was expressed in mg/g of dry fraction extract, which shows the concentration of phenolics in relation to the crude ASE extract, in equivalent phloroglucinol (PG).

### 4.6. Biological Activities Associated to Extract/Fractions of Padina pavonica

#### 4.6.1. DPPH Radical Scavenging Activity

The DPPH (2,2-diphenyl-1-picrylhdyrazyl) assay modified according to Gager et al. [5] was set up to determine the radical scavenging activity of the extract/fractions. For this method, 22 μL of extract/control was added to 200 μL of a DPPH solution (25 mg/L) prepared within 24 h. After 60 min in the dark at room temperature, the absorbance was read at 540 nm. Distilled water was used as a negative control, and ascorbic acid, α-tocopherol and 2,3-t-butyl-4-hydroxyanisole (BHA) were used as positive controls. The radical scavenging activity of the extracts was expressed as IC50 (in mg/mL), the sample concentration that results in a 50% decrease in DPPH activity. The lower the IC50, the higher the antioxidant activity. The analyses were performed in triplicate for each extract/fraction.

#### 4.6.2. Antioxidant Activity by the Ferric Reducing Antioxidant Power (FRAP)

The ferric-reducing activity was evaluated by a redox reaction between phenolic compounds in the extract and transition metal ions such as ferric ions [5]. In a microplate, 25 μL of sodium phosphate buffer (0.2 mM, pH 6.6) and 25 μL of 1% potassium ferricyanide were added to 25 μL of extract/control. After homogenization, the microplates were incubated at 50 °C for 20 min. The reaction was then stopped on ice. An initial absorbance measurement was performed at 620 nm after the addition of 25 μL of trichloroacetic acid and 100 μL of distilled water. Finally, 20 μL of iron chloride was added. After 10 min, the absorbance was measured at 620 nm. Ascorbic acid, α-tocopherol and BHA were used as positive controls. The reducing power was expressed as EC50 (mg/mL).

#### 4.6.3. Anti-Elastase Activity

The colorimetric assay was carried out by triplicate in a microplate system based on protocol reported by Gager et al. [5], which is based on the hydrolysis of N-succinyl-Ala-Ala-Ala-p-Nitroanilide (SucAla3PNA) to SucAla3 by the elastase enzyme and adapted to 96-well microplate system. Briefly, a buffer solution of Tris- HCL (179 mM, pH 8, Sigma Aldrich, Saint-Louis, MO, USA) was used to prepare a solution of SucAla3PNA (Sigma Aldrich, USA) at a concentration of 0.75 mg/mL, and to solve elastase enzyme from porcine pancreas (Sigma Aldrich, USA) at 0.34 U mL^−1^. Epigallocatechin (Sigma Aldrich, USA) was used as a positive control in a serial dilution of 0.05–1 mg/mL solved in buffer solution. Also, a blank of water was used as a negative control. For the assay, 13 μL of extracts (1 mg/mL) or control was mixed with 93 μL of buffer solution and 52 μL of SucAla3PNA. After that, microplate was incubated for 5 min at 25 °C. Then, 42 μL of diluted elastase enzyme was added to each well and mixed, and absorbances were taken at 410 nm every 48 s for 5 min. Blanks of the enzyme were prepared in the same conditions without inhibitors (control). Then, the maximum velocity (Vi) of each sample tested was calculated by software GEN5™, version 3.02 (Bio Tek, Santa Clara, CA, USA), as a principal coefficient of the linear wave bulker. Percentage of inhibition (PI) was calculated with the following formula:

PI (%) = [Vicontrol − Visample)/Vicontrol] × 100

#### 4.6.4. Anti-Tyrosinase Activity

Depigmentation, considered as an anti-ageing activity, was evaluated thanks to the assessment of the tyrosinase inhibition activity of extracts. The protocol was inspired by Gager et al. [30]. A phosphate buffer was prepared at pH 6.5 and 0.1 M by mixing 4.117 g of Na_2_HPO_4_ and 8.639 g of NaH_2_PO_4_. The second solution, corresponding to the substrate, consists of a 1.694 mM L-tyrosine solution solubilized in distilled water. A volume of 2.5 mL of this solution was then added to 2.5 mL of phosphate buffer and 2.25 mL of distilled water to obtain the L-tyrosine reagent. Then, the enzymatic solution was at a concentration of 317.71 U.mL^−1^ of tyrosinase. In the microplate, 20 μL of extract or control were added with 243 μL of L-tyrosine reagent and the mixture was incubated at 25 °C for 5 min. Finally, 42 μL of enzymes were added and a kinetic was performed at 475 nm, always at 25 °C, for 5 min. A blank was made for each extract replacing substrate with buffer. Kojic acid was used as a positive control. The percentage of inhibition of the enzyme was calculated in the same way as for the elastase inhibition and compared with that of the positive control.

### 4.7. Statistical Analysis

The statistical analysis was performed using Origin Pro (version 2024). All laboratory experiments were conducted in triplicate, and the results were expressed as the mean ± standard deviation (SD). The homogeneity of variances was tested using Bartlett’s test at a significance level of 0.05. When homogeneity was confirmed, a one-way ANOVA was carried out, followed by Tukey’s post-hoc test for multiple comparisons. However, if the assumption of homogeneity of variances was violated, indicating that the data did not meet the ANOVA assumptions, a non-parametric Kruskal–Wallis test was applied with a 95% confidence level. When significant differences were detected, non-parametric multiple comparison tests were performed to further investigate the differences between groups.

## 5. Conclusions

Our study highlights the significant potential of the brown macroalga *P. pavonica*, a widely abundant brown seaweed found in Djibouti, as a potential source of active ingredients for the cosmetic and/or cosmeceutical industry. We successfully isolated phlorotannin-rich fractions that exhibit strong biological activity. For this, we applied sustainable and environmentally friendly extraction and purification processes, designed to minimize environmental impact. The eco-friendly extraction methods, such as SPE_WE50 and FC_EtOH20, alongside the conventional LLE_FAE process, demonstrated high concentrations of active phlorotannins, with isolated and identified phlorotannins that depend on the purification procedure. The NMR and LC-MS analysis of these fractions confirmed that the purification methods strongly influenced the selectivity of phlorotannins, particularly about their degree of polymerization. LLE permitted to obtain a large variety of compounds, which is not the case for the other two processes. The two eco-friendly procedures permitted to generate active fractions with a time and solvent-used gain. The most phlorotannin-rich fractions were found to exhibit the highest levels of antioxidant and anti-elastase/tyrosinase activity, establishing a direct relationship between the degree of polymerization and bioactivity. These results underline the relevance of the brown macroalga *P. pavonica* as a valuable source of cosmetic ingredients, offering strong bioactive potential while supporting sustainable practices in the industry.

## Figures and Tables

**Table 1:** Yields (% DW), total phenolic content (mg/g DW), and antioxidant activity screening of crude extracts (ASE) and purified fractions (LLE, SPE, and FC), assessed by radical scavenging activity (DPPH; IC50 in mg/mL) and reducing power assay (FRAP; EC50 in mg/mL). Each value represents the mean ± SD (*n* = 3). The fractions selected for their activities and the most interesting activity test values are highlighted in bold.

Method	Yield (% Relative to the Initial Dry Mass)	TPC mg PGE/g DW	DPPH IC50 mg/mL	FRAP EC50 mg/mL
ASE	18.2	94.9 ± 1.5	0.29 ± 0.01	0.25 ± 0.012
**LLE_FAE**	9.2	**155.1 ± 1.3**	**0.03 ± 0.00**	**0.11 ± 0.04**
FC_W	39.1	53.2 ± 1.5	2.14 ± 0.2	1.41 ± 0.12
**FC_EtOH20**	**3.3**	**132.1 ± 1.7**	**0.06 ± 0.01**	**0.12 ± 0.03**
FC_EtOH50	3.2	71.8 ± 3.8	0.83 ± 0.07	1.11 ± 0.13
FC_EtOH75	8.9	56.1 ± 2.2	1.31 ± 0.07	1.40 ± 0.07
FC_EtOH100	4.0	90.6 ± 5.9	0.96 ± 0.12	1.01 ± 0.12
SPE_W	38.8	40.8 ± 2.5	1.85 ± 0.51	0.49 ±0.10
**SPE_WE50**	**13.2**	**129.4 ± 1.5**	**0.10 ± 0.09**	**0.15 ± 0.04**
SPE_E	10.3	65.7 ± 2.5	0.16 ± 0.09	0.22 ± 0.09
SPE_ED	3.1	77.7 ± 4.5	2.11 ± 0.75	3.10 ± 0.65
SPE_D	2.1	31.7 ± 2.5	2.30 ± 0.88	2.50 ± 0.75
Vitamin C	/	/	**0.02**	**0.08** ± 0.01
BHA	/	**/**	**0.01**	**0.09** ± 0.01

**Table 2:** Identification of signals on combined ^1^H-^13^C HMBC NMR spectra of the purified fractions (solvent MeOD), enabling the determination of categories of phlorotannins, adapted from the literature data [23,46,47]. 2D NMR spectra are provided as Supplementary Materials (Figure S1).

Type of Purification	Chemical Shifts in the ^1^H Dimension δ (ppm)	Signal on the ^1^H	Chemical Shifts in the ^13^C Dimension δ (ppm)	Signal on the ^13^C	Type of Phlorotannins
LLE-FAE	5.75	Singulet	92–95	Methine zone (C-H of the aromatic ring)	Phloroglucinol
	5.8–6.2	Massif of peaks and doublets	100–105	Aryl-Aryl carbons	Fucol
	5.8–6.2	Massif of peaks and doublets	125–130	Diaryl-ether carbons	Fucophlorethol
	5.8–6.2	Massif of peaks and doublets	145–150	Hydroxyl functions	Fuhalol
	5.8–6.2	Massif of peaks and doublets	150–165	Phenolic carbons	
SPE-WE50	5.75	Singulet	92–95	Methine zone (C-H of the aromatic ring)	Phloroglucinol
	5.8–6.2	Massif of peaks	125–130	Diaryl-ether carbons	Fucophlorethol
	5.8–6.2	Massif of peaks	162–165	Phenolic carbons	
FC-EtOH20	5.75	Singulet	92–95	Methine zone (C-H of the aromatic ring)	Phloroglucinol
	5.8–6.2	Not defined signals	125–130	Diaryl-ether carbons	Fucophlorethol
	5.8–6.2	Not defined signals	162–165	Phenolic carbons	

**Table 3:** LC-ESI-QTOF-MS characterization of purified fractions of phlorotannins from *Padina pavonica* obtained in negative ionization mode. The identification of compounds was done in combining LC-MS data and reference to the literature data [35,45].

N°	Name	RT	Formula	MW (in Da)	[M − H]^−^ *m*/*z*	Type of Purification
1	Phloroglucinol (monomer)	1.11	C_6_H_6_O_3_	126.0317	125.0255	LLE_FAE
2	Phlorethol/Fucol (dimer)	1.09	C_12_H_10_O_6_	250.0491	249.0415	LLE_FAE
3	Fucophlorethol (trimer)	1.08	C_18_H_14_O_9_	375.5178	374.2470	LLE_FAE
		1.08	C_18_H_14_O_9_	375.2526	374.2599	FC_EtOH20
		1.08	C_18_H_14_O_9_	375.2511	374.2592	SPE_WE50
4	Fuhalol (dimer)	0.85	C_12_H_10_O_7_	266.1565	265.1499	LLE_FAE

**Table 4:** Known molecules that may correspond to the analyses performed in negative and positive ionisation modes of LC-ESI-QTOF-MS from LLE-FAE, SPE_WE50, FC_EtOH20 of the brown macroalga *Padina pavonica*. Formula were proposed by the software MassHunter Qualitative Analysis version B.06.00., and also with the using of the on-line MarinLit and ChemSpider databases.

N°	Name	Formula	MW (in Da)	*m*/*z*	Type de Purification	Polarity	RT
1	Arachidonic acid or Dictyotadiol	C_20_H_32_O_2_	304.2402	303.2347	LLE-FAE	negative	1.05
2	Dolastanes (Diterpene)	C_20_H_30_O_2_	302.2265	301.2193	LLE-FAE	negative	1.06
3	Hexyl 2F-furoate or (+)-epiloliolide (norisoprenoid)	C_11_H_16_O_3_	196.1099	197.1171	FC_EtOH20	positive	1.15
4	Licoflavone C or Asperterphenylcin B	C_20_H_18_O_5_	337.3345	338.3406	LLE-FAE	positive	1.08
5	Asperbiphenyl	C_24_H_30_O_6_	414.4936	415.2104	LLE-FAE	positive	1.11
		C_24_H_32_O_6_	414.2066	415.2126	SPE_WE50	positive	1.02
		C_24_H_30_O_6_	414.2051	415.2116	FC_EtOH20	positive	1.11
6	Cymatherol B (polycyclic oxylipins)	C_18_H_28_O_4_	308.1988	309.2059	FC_EtOH20	positive	1.20
		C_18_H_28_O_4_	308.1988	307.1909	SPE_WE50	negative	1.11
7	Phtalonic acid	C_9_H_6_O_5_	194.0579	193.0147	FC_EtOH20	negative	0.83
		C_9_H_6_O_5_	194.0579	193.0147	SPE_WE50	negative	0.83
8	Mannitol	C_6_H_14_O_6_	182.0790	181.0716	SPE_WE50	negative	2.30
		C_6_H_14_O_6_	182.0790	181.0717	LLE_FAE	negative	2.30
		C_6_H_14_O_6_	182.1760	181.0721	FC_EtOH20	negative	2.30
9	Penicitide B or Carnosol	C_18_H_34_O_5_	330.4754	329.2327	SPE_WE50	negative	1.16

		C_20_H_26_O_4_					
10	4-[(E)-2-Carboxyvinyl]-2-methoxyphenyl β-D-glucopyranosiduronic acid	C_20_H_34_O_6_	370.9217	369.2273	SPE_WE50	negative	1.19
11	Peonidin 3-O-sambubioside-5-O glucoside	C_33_H_41_O_20_	757.2146	758.2218	SPE_WE50	positive	1.02
		C_33_H_41_O_20_	757.2146	758.2218	FC_EtOH20	positive	1.03
12	Identification in progress	C_24_H_47_ClO_7_	482.3024	481.2948	LLE_FAE	negative	1.18

## Data Availability

The data sets generated during and/or analyzed during the current study are available from the corresponding author upon reasonable request.

## Supplementary Materials

The following supporting information can be downloaded at: https://www.mdpi.com/article/10.3390/md23010015/s1, Figure S1: ^1^H-^13^C HMBC NMR spectra of the purified fractions (solvent: MeOD) from the brown macroalga *Padina pavonica*. Horizontal axis: ^1^H dimension; vertical axis: ^13^C dimension. P: phlorotannins area (circa 5.8–6.2 ppm in ^1^H. PG: phloroglucinol singlet (5.75 ppm in ^1^H); Figure S2: Chromatograms of the three purified fractions from the brown macroalga *Padina pavonica* and obtained using Accurate-Mass Q-ToF LC-MS analysis; Figure S3: LC-ESI-QTOF-MS characterization of purified fractions. Mass spectrum of elucidated phlorotannins from the brown macroalga *Padina pavonica*: phloroglucinol, diphlorethol/difucol, fucophlorethol, and Bifuhalol. Their retention times (RT) and ionization modes are summarized in Table 2. Figure S4: Mass spectrum of a chlorinated compound (compound 12, Table 4) with a [M − H]^−^ *m*/*z* of 481.2948 and a retention time of 1.18 min, extracted from the brown macroalga *Padina pavonica* and obtained using Accurate-Mass Q-ToF LC-MS analysis.
