# Supplementary material for: Eco-Friendly Extraction of Phlorotannins from Padina pavonica: Identification Related to Purification Methods Towards Innovative Cosmetic Applications
Source: Mar Drugs. 2024 Dec 28;23(1):15. doi: 10.3390/md23010015 (PMC11766817; doi:10.3390/md23010015)
Supplement: Supplementary file 1 [file marinedrugs-23-00015-s001.zip › marinedrugs-3346364-Supplementary.pdf]

marine drugs

MDPI

Article

Eco-friendly extraction of phlorotannins from *Padina pavonica*: identification related to purification methods towards innovative cosmetic applications

Moustapha Nour [1,2], Valérie Stiger-Pouvreau [1,*], Alain Guenneguez[1], Laurence Meslet-Cladière [3], Stéphane Cérantola [4], Ahmed Ali[2], Gaelle Simon [4], Abdourahman Daher [2] and Sylvain Petek [1]

[1] Univ Brest, IRD, CNRS, Ifremer, LEMAR, IUEM, F-29280 Plouzane, France; moustapha.nouribrahim@univ-brest.fr (M.N.); valerie.stiger@univ-brest.fr (V.S.-P.) ; alain.guenneguez@ird.fr (A.G.); sylvain.petek@ird.fr (S.P.)
[2] Centre d'Études et de Recherche de Djibouti, Institut des Sciences de la Vie ISV, Route de l'aéroport, Djibouti; abd_daher@yahoo.fr (A.D.) ; aafarhan@hotmail.fr (A.A)
[3] Univ Brest, INRAE, Laboratoire Universitaire de Biodiversité et Écologie Microbienne, F-29280, Plouzane, France; laurence.meslet@univ-brest.fr (L.M.-C.)
[4] Univ Brest, Plateforme RMN-RPE, F-29238 Brest, France; stephane.cerantola@univ-brest.fr (S.C.) ; gaelle.simon@univ-brest.fr (G.S.)
* Correspondence: valerie.stiger@univ-brest.fr (V.S.-P.)

Citation: To be added by editorial staff during production.

Academic Editor: Firstname Lastname

Received: date
Revised: date
Accepted: date
Published: date

Copyright: © 2024 by the authors. Submitted for possible open access publication under the terms and conditions of the Creative Commons Attribution (CC BY) license (https://creativecommons.org/licenses/by/4.0/).

SUPPLEMENTARY MATERIALS

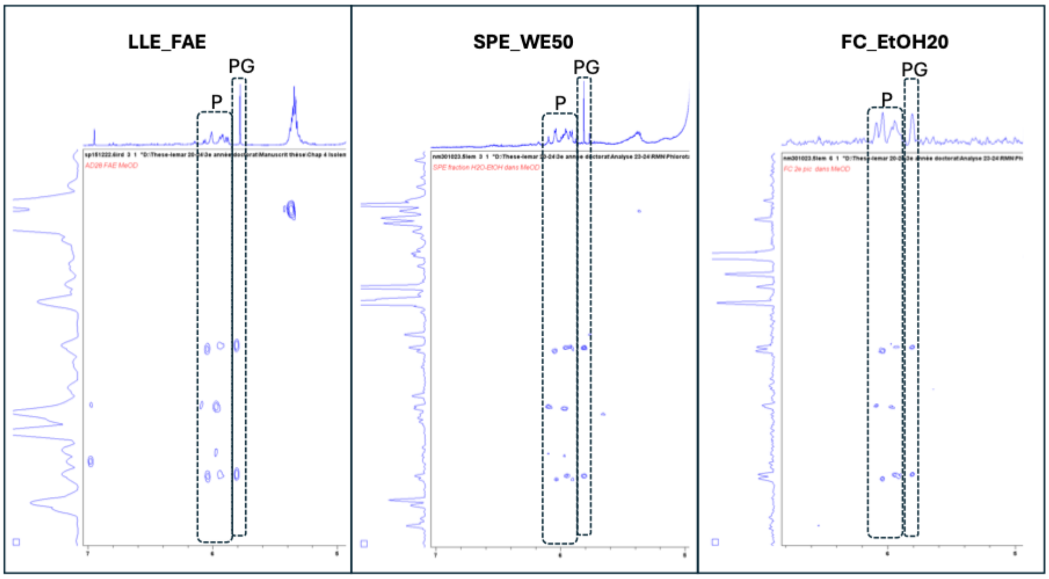

Figure S1. ^{1}H-^{13}C HMBC NMR spectra of the purified fractions (solvent: MeOD) from the brown macroalga *Padina pavonica*. Horizontal axis: ^{1}H dimension; vertical axis: ^{13}C dimension. P: phlorotannins area (circa 5.8–6.2 ppm in ^{1}H). PG: phloroglucinol singlet (5.75 ppm in ^{1}H).

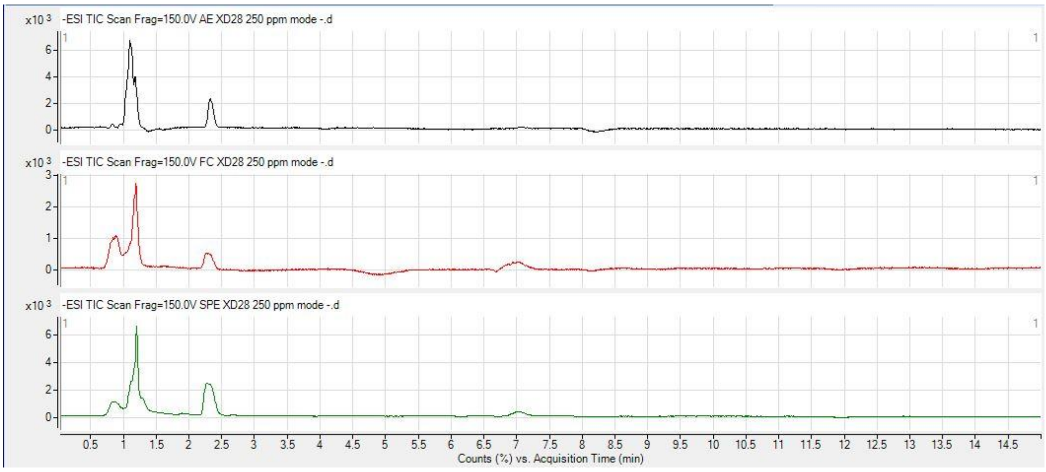

Figure S2. Chromatograms of the three purified fractions from the brown macroalga *Padina pavonica* and obtained using Agilent 6530 Accurate-Mass Q-ToF LC-MS analysis. LLE_FAE (top), FC_EtOH20 (middle), SPE_WE (bottom)

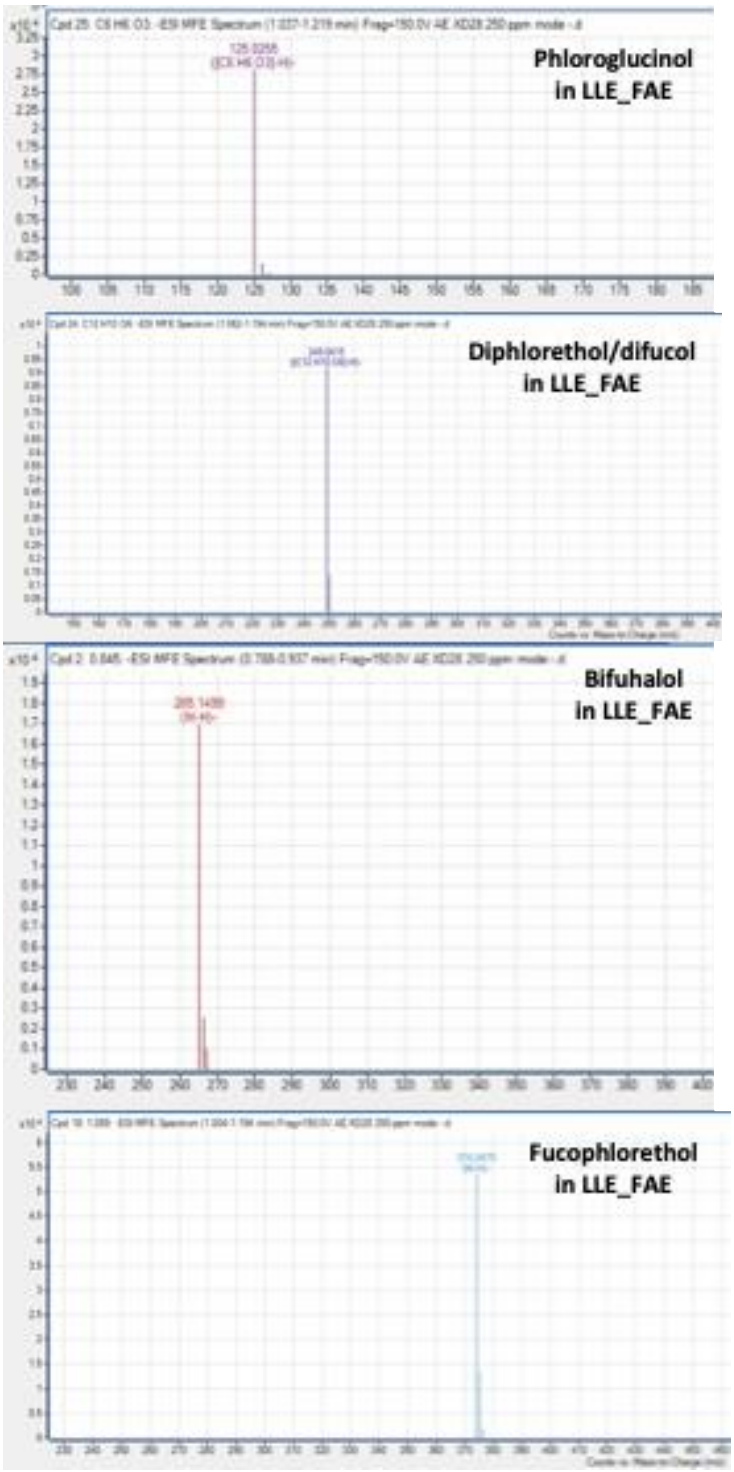

Figure S3. LC-ESI-QTOF-MS characterization of purified fractions. Mass spectrum of of elucidated phlorotannins from the brown macroalga *Padina pavonica:* phloroglucinol, diphlorethol/difucol, fucophlorethol, and Bifuhalol. Their retention times (RT) and ionization modes are summarized in Table 2.

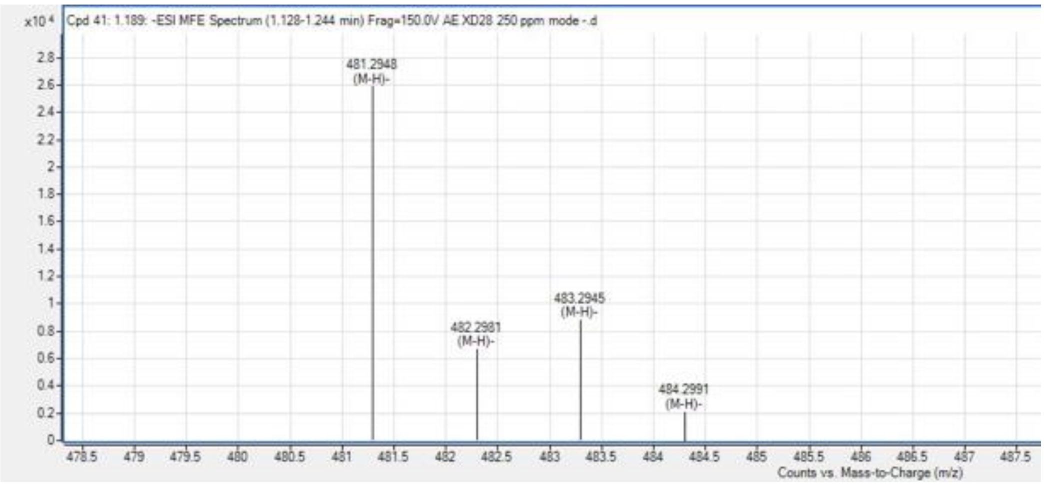

Figure S4. Mass spectrum of a chlorinated compound (compound 12, **Table 4**) with a $[M\text{-}H]^-$ *m*/*z* of 481.2948 and a retention time of 1.18 min, extracted from the brown macroalga *Padina pavonica* and obtained using Agilent 6530 Accurate-Mass Q-ToF LC-MS analysis.
